# Effects of Non-Starch Polysaccharides on Inflammatory Bowel Disease

**DOI:** 10.3390/ijms18071372

**Published:** 2017-06-27

**Authors:** Ying Nie, Qinlu Lin, Feijun Luo

**Affiliations:** 1 Laboratory of Molecular Nutrition, College of Food science and Engineering, National Engineering Laboratory for Deep Processing of Rice and Byproducts, Central South University of Forestry and Technology, Changsha 410004, China; ny198722@hotmail.com (Y.N.); linqinlu@hotmail.com (Q.L.); 2 Department of Animal Science and Technology, College of Hunan Biological and Electromechanical Polytechnic, Changsha 410128, China

**Keywords:** non-starch polysaccharide (NSP), inflammatory bowel disease (IBD), intervention, mechanism, SCFAs, gut microbiota, immune system, pro-inflammatory cytokines

## Abstract

The incidence of inflammatory bowel disease (IBD) has increased considerably over the past few decades. In the present review, we discuss several disadvantages existing in the treatment of IBD and current understandings of the structures, sources, and natures of various kinds of non-starch polysaccharides (NSPs). Available evidences for the use of different sources of NSPs in IBD treatment both in vitro and in vivo are analyzed, including glucan from oat bran, mushroom, seaweed, pectin, gum, prebiotics, etc. Their potential mechanisms, especially their related molecular mechanism of protective action in the treatment and prevention of IBD, are also summarized, covering the anti-inflammation, immune-stimulating, and gut microbiota-modulating activities, as well as short-chain fatty acids (SCFAs) production, anti-oxidative stress accompanied with inflammation, the promotion of gastric epithelial cell proliferation and tissue healing, and the reduction of the absorption of toxins of NSPs, thus ameliorating the symptoms and reducing the reoccurrence rate of IBD. In summary, NSPs exhibit the potential to be promising agents for an adjuvant therapy and for the prevention of IBD. Further investigating of the crosstalk between immune cells, epithelial cells, and gut microorganisms in addition to evaluating the effects of different kinds and different molecular weights of NSPs will lead to well-designed clinical intervention trials and eventually improve the treatment and prevention of IBD.

## 1. Introduction

Inflammatory bowel disease (IBD) is mainly characterized by intermittent inflammation in the gastrointestinal tract [1], which can be subdivided into ulcerative colitis (UC) and Crohn’s disease (CD), and its incidence has increased considerably over the past few decades [2,3]. The common clinical features of IBD include persistent diarrhea, vomiting, hematochezia, unintentional weight loss, and pain. UC is a chronic non-specific inflammatory bowel disease. UC occurs only in the large bowel, and the inflammation is confined to the mucosa, while CD may occur anywhere in the digestive tract, from mouth to anorectum, and it affects the entire bowel wall to form abscesses and fistulas in the skin or internal organs [4]. The increasing prevalence of IBD in Western countries has incited growing attention and efforts worldwide to find new effective strategies for IBD treatment.

Many drugs used as treatment options for IBD in clinical practice were reported with adverse effects. Antibiotics have been noted for their severe side effects, causing bacterial resistance and dysbiosis in the gut microbiome. Aminosalicylates (PPARγ agonist) exert only a moderate effect on CD, but also causing headaches and nausea [5]. Corticosteroids, also used in other anti-inflammatory treatments, tend to lead to osteoporosis, hypertension, obesity, type 2 diabetes and aggravating gastrointestinal ulcers [6]. Other drugs, such as immunosuppressive agents like 6-mercaptopurine and some biological antibodies like Infliximab (anti-TNFα), also have shown adverse reactions such as mood disturbances, dyspepsia, and sleeping problems, etc. [7]. Besides these adverse reactions or side effects, these drugs are only effective for remitting the disease rather than offering a cure. Medical scientists thus are dedicated to develop a new adjuvant therapy strategy especially at the early stage of IBD, which may involve a food-source natural product as dietary modifications have showed the potential to help induce remission of the disease [8]. Non-starch polysaccharides (NSPs) are an ideal selected target, as proven by many different studies both in vivo and in vitro. The purpose of the present study is to review the literature involving the effects of NSPs on the treatment of IBD and its possible mechanism of action.

## 2. Structures, Compositions, and Sources of NSPs

Non-starch polysaccharides, together with resistant starch and lignin, form the whole of dietary fiber, which has been reported to exhibit multiple biological functions that are able to withstand a series of diseases, including cancer, type 2 diabetes, hyperlipemia, cardiovascular diseases (CVD), and obesity [9,10]. The major components of NSP are cellulose, hemicellulose, pectin, β-glucan, pentosane, and xylan, which are all resistant to hydrolyzation by any endogenous digestive enzymes of non-ruminant animals. It is now commonly agreed that the vast majority of edible NSP leaves the small intestine almost intact, and is fermented by the commensal microflora in the large intestine (caecum and colon). NSP is generally classified into water soluble or insoluble types. Plants generally contain both soluble and insoluble NSPs, however, their proportion varies according to the type and maturity degree of the plant [11]. 

Insoluble NSPs mainly consist of cellulose and hemicellulose. Cellulose is composed of up to 10,000 d-glucoses binding with β-l,4-glycocisidic bonds. This is the base of the micro-fibril structure and the reason why cellulose is an insoluble polysaccharide. Hemicelluloses constitute a series of heterogenic monosaccharides and most of them can dissolve in alkali solutions but not in water. Cereal hemicellulose contains mainly arabinoxylans and arabinogalactans, which are assembled mainly with xylans or galactans as backbone and arabinose or pentosans as side chains. 

**Figure 1:**
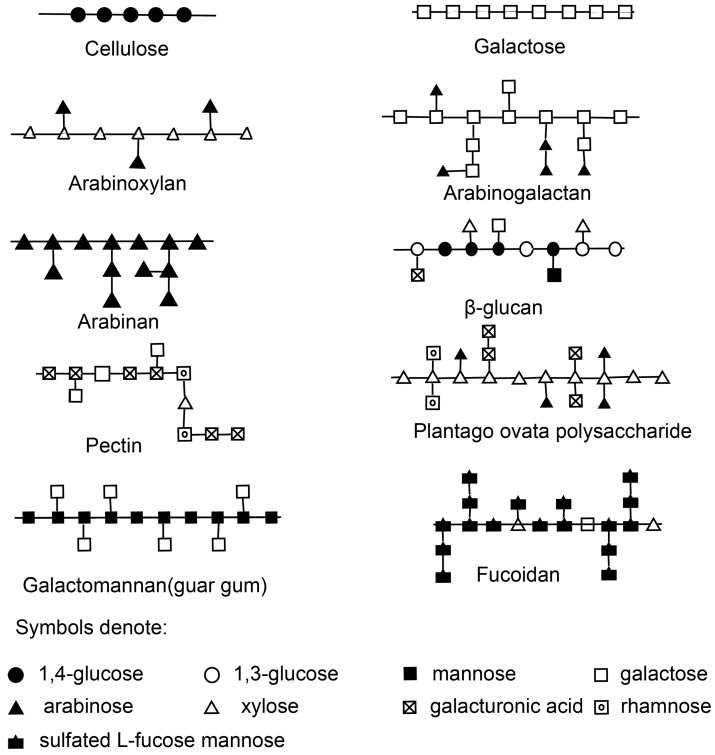
Structures of typical non-starch polysaccharides.

Most other types of NSPs are able to dissolve in water. The endospermic cell wall polysaccharides of wheat and rye are mainly water-soluble [12]. In barley and oats, β-l,3- and β-l,4-linked water soluble β-glucan are the predominant constituents, where they account for about three quarters of the cell wall dry matter. Glucan is also commonly found in the fungal kingdom, such as in *Ganoderma* and *Lentinula edodes*, whose backbones are composed mainly of mixed α- and β-d-glucan or pure β-d-glucan with heterosaccharide side chains of xylose, mannose, galactose, or uronic acid. These can be both water soluble or insoluble [13]. NMR spectroscopy was used to analyze the glucan structure of seaweed polysaccharide, and it was found to be composed of a β-1,3-glucan backbone and about 20% of β-1,6-glucan side chains [14]. The insoluble parts are usually structural components of the cell wall and are crosslinked to other polysaccharides like chitin or to proteins. The soluble fraction holds about 20–50% of the total glucan while the insoluble part takes the share between 50% and 80% [15]. Seaweed polysaccharides include alginates, carrageenans, and agar, and they differ in composition from one another. Alginates typically consist of 200–1000 d-mannuronic acid- and l-guluronic-acid residues. Carrageenan and agar are sulphated galactans based on linear chains of galactose residues [16]. Pectins dissolve mainly in hot water and form a gel when the temperature drops, whose main monomeric component is d-galacturonic acid with interruptions of rhamnose or galactose. Plant-secreted gums such as gum arabic and karaya have highly branched molecular structures and contain several monosaccharide residues as well as uronic acids. Mucilages possess a high hydrophilic ability and are found in special mucilage cells of the outer layer of seeds of the plantain plant. The molecule of mucilage from *Plantago ovata* (*ispaghula*) includes 30–35 residues, consisting of β-l,4- and β-l,3-linked d-xylose residues as the backbone, and arabinose, rhamnose, and galacturonic acid as side chains. There are galactomannan polysaccharides in the endosperm cell walls of leguminous seeds, which are usually referred to as guar gum and locust bean gum. The structures of the most typical NSPs are illustrated in Figure 1.

As described above, the major source of NSPs are plants, especially the endospermic cell wall of multiple kinds of seeds, fungi, and algae [17], and they form a rigid structure surrounding plant cells and thus avoid being hydrolyzed by the digestive enzymes in the human small intestine, but are instead fermented by microorganisms to produce short chain fatty acids (SCFAs) beyond the small intestine. Various types of NSPs possess different properties and therefore exert different health impacts on intestinal diseases. Potential benefits include reducing diarrhea and constipation, promoting tissue healing, enhancing immune ability, relieving inflammation, producing SCFAs, and reducing the absorption of carcinogens. Through these means, NSPs can alleviate the symptoms of IBD patients and further decrease the risk of colorectal cancer (CRC) [18,19,20].

## 3. Effects of NSPs on IBD

### 3.1. Oat Bran Glucan

Non-starch polysaccharides were first introduced as a clinical treatment for human UC in 1978 by Davies [21], in which oat bran was applied. However, the main conclusion drawn from this trial was that high bran intake is of less value in maintaining clinical remission in patients with UC compared to the drug group. In their study, they overlooked the difference of UC patients; oat bran may be effective for patients with mild symptoms, but it may be useless for patients with severe symptoms. In addition, the dose of oat bran (25 g/day) was insufficient compared to other studies. 22 quiescent UC patients with about 1 year from last relapse for 3 months were daily administrated with 60 g oat bran (main composition is 1,3- and 1,4-glucan). The results showed that the concentration of fecal butyrate increased by about 36%, and abdominal pain and gastrointestinal reflux improved significantly after the oat bran intervention. Moreover, the intervention did not cause any increase of the disease relapse or GI complaints [22], which suggests the feasibility of long-term maintenance therapy in UC using oat bran.

There are several studies on oats β-glucan in animal IBD models. β-glucan from oat (purity 75%) with high (G1) and low (G2) molecular weight was used to evaluate the anti-inflammatory and antioxidant effects in lipopolysaccharide (LPS)-induced chronic enteritis. Results revealed that supplementation with both glucan fractions led to a significant reduction of blood leucocytes, but only G1 reduced the lipid peroxidation in the enteritis model [23]. We have also investigated the protective effect of oat β-glucan (βG) on the dextran sulfate (DSS)-induced colitis of mice and its possible molecular mechanism. We found that the oral administration of βG significantly alleviated clinical symptoms of mice, which included decreasing the disease activity index (DAI), weight loss, diarrhea, and increasing the colon length. Hematoxylin-eosin (HE) staining showed that βG ameliorated the DSS-induced histological damage and reduced the infiltration of inflammatory cells. Further investigations showed that the oral administration of βG decreased the levels of myeloperoxidase (MPO), nitric oxide (NO) and malondialdehyde (MDA), as well as downregulated the expressions of pro-inflammatory factors in the colonic tissues [24]. Other publications also suggested that oat glucan also can exert a protective effect on other intestinal diseases and symptoms, especially celiac disease and constipation [25]. 

### 3.2. Mushroom Glucan 

Mushrooms have been applied as medicinal therapy since ancient times, especially in Asian countries. About 20 species out of more than 2000 kinds of mushrooms are used for adjutant therapy or for the prevention of inflammation. Forland et al. [26] proved that the oral administration of AndoSan™, a mixed extract from basidiomycetes mushrooms, alleviated the inflammatory symptoms of IBD patients. They found a significant decrease of pro-inflammatory cytokines in plasma after 12 days intake of AndoSan™. Calprotectin, a vital inflammatory marker in the feces of the UC patients, was also decreased in the study. Another study suggested that Chaga mushroom extract can protect DNA against the oxidative damage in the lymphocytes of IBD patients [27]. AndoSan™ has also been applied in another randomized placebo-controlled study; however, these 50 patients exhibited active UC and CD, and only limited anti-inflammatory effects were observed in these patients [28].

Our latest work evaluated the anti-inflammation effect of *lentinus edodes* β-glucan (βG) and its molecular mechanism. Body weight, DAI, and inflammatory symptoms were all improved after the oral administrations of βG in mice. β-glucan decreased the contents of MDA and MPO of colonic tissues, and downregulated the expression of iNOS, TNF-α, IL-1β, and IL-6 in the colonic tissues of mice. In a DSS-induced colitis model and LPS-stimulated RAW264.7 cell model, βG inhibited the expression of pro-inflammatory factors and suppressed the phosphorylation of Elk-1 at Ser84 as well as the phosphorylation of PPARγ at Ser112. This suggests that βG participates in the anti-inflammatory function via a complex signal network. [29]. Other investigators have demonstrated that the oral administration of glucan from *P. pulmonarius* reduced the intestinal inflammation and DSS-induced symptoms in mice presenting colitis. This glucan also may prevent colorectal cancer (CRC) incidence along with IBD and reduce the expression of the proliferating-associated marker, proliferating cell nuclear antigen (PCNA) in adenocarcinomas [30]. Another study showed that lentinan may prevent CRC in susceptible UC animals by downregulating the expression of P4501A2, which was modulated by TNF-α, and changing the DNA-binding activity of NF-κB [31]. Carcinogenesis and inflammation share some common pathways and inflammation is closely related with carcinogenesis. For example, the activation of AP-1, NF-κB, and MAPK are involved in carcinogenesis and inflammation. Several publications showed that glucan extracted from mushrooms can inhibit the proliferation of human colorectal cancer cells and lead to the apoptosis of tumor cells. In China, βG has been used as an adjuvant drug for cancer patients in clinic [32,33,34,35]. Our paper also provides new evidence of glucan reducing inflammatory cytokine expressions in cell and animal inflammation models, suggesting that NSPs may be used as inhibitors of inflammation in the prevention or adjuvant therapy of IBD.

### 3.3. Seaweed-Derived β-Glucan

A study shows β-glucan obtained from *Laminaria hyperborea* and *Laminaria digitata* can both significantly decrease the expression of Th17-associated cytokines (IL-17a, IL-17F, and IL-22) as well as receptor IL23R and IL-6, with no alteration to the T regulatory cell (TREG)–related targets [36]. Given that the Th17 inflammatory response has been regarded as a main target contributing to the underlying pathology of IBD, seaweed-derived glucan showed a great potential to be applied in the neoadjuvant therapy of the disease. This team studied further whether that algal polysaccharide laminarin, a β-(1–3 and 1–6)-linked glucan, exerts a protective effect on DSS-challenged pigs. The results exhibited improved body-weight loss and clinical symptoms, colonic Enterobacteriaceae, and a reduction of colonic IL-6 mRNA expression level [37]. However, no clinical trial in humans has been conducted so far.

### 3.4. Other Glucan

Similar to oats glucan, bacterial β-(1,3)-glucan also shows a protective effect on IBD in mice from body weight data, disease score, and histological score. The recruitment of macrophages and the expression of pro-inflammatory cytokines (IL-1β, IL-6, and IL-17) were obviously decreased in the colon tissues of mice after bacterial glucan supplement. It also induced the recovery of Tregs, repaired the functional defects of natural killer (NK) cells, and modulated the abnormal IgA production in DSS-induced colitis mice [38]. According to Jin et al. [39], the root of *Angelica sinensis* (Oliv.) harbors plenty of heterogenic polysaccharides of mainly α-1,3-glucan, α-1,6-glucan, and other linear α-glucan, which has been proven to have immune-modulatory and anti-inflammatory properties. Comparing the effect of β-1,3 and 1,6-d-glucan, β-hydroxy-β-methyl-butyrate (HMB), and levamisole on IBD in a canine model, the results suggested that the glucan was the best and reacted the fastest among the three agents [40]. Taken together, in spite of the sources of glucan, many evidences suggest that glucan can provide beneficial effects on IBD and IBD-related symptoms. 

### 3.5. Fucoidan 

Fucoidans are acidic and sulfated macromolecules composed of L-fucose along with several other oligosaccharides such as mannose, galactose, and xylose. They are usually extracted from brown algae such as *Fucus vesisulosus* and the sporophyll of *Undaria pinnatifida*. Fucoidans can provide health benefits such as antioxidant, anti-inflammatory, anti-allergic, antitumor, anti-viral, and anti-hepatopathy potentials [41]. The oral administration of high purity of fucoidan significantly ameliorated colitis-associated symptoms, and histological examination showed the obvious reduction of crypt architecture and goblet cells, as well as the infiltration of immune cells and oedema. These protective effects were validated by decreasing expressions of 15 pro-inflammatory cytokines in the colonic tissues, which implied that fucoidans could be a treatment option for IBD [42]. O’Shea et al. [37] suggested that a diet containing fucoidan is a possible nutritional therapy for UC patients based on their observations of the treatment ameliorating diarrhea, pathology score, and decreasing IL-6 expression in DSS-induced colitis of pigs. Fucoidan also inhibited fat accumulation via decreasing *aP2* and *PPARγ* gene expressions, which led to the decreased expressions of inflammation-related genes [43,44]. These evidences suggest that fucoidan may exert beneficial effects on the alleviation of IBD-related symptoms.

### 3.6. Polysaccharides from Plantago ovata Seeds 

One hundred and five patients with stable remissive UC were randomly divided to receive either *Plantago ovata* seeds or mesalazine (5-aminosalicylic acid) with the same dose. After a half year, the recurrence rate of different groups had no significant difference. These surprising results showed that *Plantago ovata* seeds exhibit therapeutic potential of UC. [45]. HLA-B27 transgenic rats were fed with 5% *Plantago ovata* seeds for 13 weeks to evaluate the effects of its anti-inflammatory property in the colitis model. The intestinal cytoarchitecture was obviously ameliorated and the colonic inflammation was inhibited by decreasing the expressions of pro-inflammatory cytokines including NO, leukotriene B(4), and TNF-α. Rats with supplementation produced a higher number of SCFAs than that of control group [46]. The team confirmed further that 5% *Plantago ovata* seeds polysaccharides have a similar protective effect on trinitrobenzenesulfonic acid (TNBS)-induced colitis [47].

### 3.7. Wheat Bran and Barley

Arabinoxylans are stored in whole grain cereals with high quantities and their supplementation are reported have beneficial biological effects. Germinated barley (GB) was used in a clinical trial, in which 59 UC patients in a remission state were divided into two groups; 37 individuals in the control group received a conventional drug for one year and the other 22 patients in the GB group received a conventional drug plus 20 g GB daily. GB significantly ameliorated the disease activity index and reduced the recurrence rate, meanwhile no side effects were observed in the GB group [48]. Neyrinck et al. [49] reported the prebiotic effects of wheat arabinoxylan. They found that the abundance of *Bacteroides* and *Roseburia spp*. as well as bifidobacterial was increased after wheat arabinoxylan supplementation, and the gut barrier function was strengthened with lower serum inflammatory markers. In an eight-week clinical trial, 19 UC patients intaking wheat bran (WB) and resistant starch (RS) increased prebiotic effects, which normalized gut transit, lowered the proportions of *Akkermansia muciniphila,* and increased diversity within the *Clostridium cluster XIVa* compared to the control group [50].

### 3.8. Prebiotics

According to the definition of the 6th Meeting of The International Scientific Association for Probiotics and Prebiotics (ISAPP) (2008), prebiotics are a selectively fermented ingredient that results in specific changes of the gastrointestinal microbiota (including the composition and activity), thus conferring benefit upon host health. Among prebiotics, oligofructose supplementation increases the abundance of *bifidobacteria* in the intestine and improves the mucosal barrier function of the intestine by reducing the expressions of pro-inflammatory cytokines [51]. Purified xylooligosaccharides were used in a simulation of an intestine fermentation experiment, and are involved with human fecal microbiota. Xylooligosaccharides result in a decreased pH, increased production of SCFAs, and an increased number of *Bifidobacterium*, *Lactobacillus*, and *Escherichia coli*, suggesting the intestine-health-promoting effect of xylooligosaccharides [52]. 

There were several reports on the intervention using probiotics. In one double-blind and placebo-controlled trial, 22 patients with active UC were enrolled to evaluate the effect of VSL#3, a probiotic strain. This showed that the treatment of UC patients with VSL#3 had a beneficial effect, which included enhanced function of intestinal dendritic cells, increased expressions of regulatory cytokines, and reduced expressions of pro-inflammatory cytokines as well as toll-like receptor (TLR) [53]. Other investigators used 27 IBD dogs to assess the effects of chondroitin sulfate and several prebiotics including resistant starch, β-glucan, and mannaoligosaccharides on IBD lasting six months. After treatment, the histologic score of dogs decreased 1.53-fold in the supplement group [54]. 

Several prebiotics or probiotics trials were used to study irritable bowel syndrome (IBS). Three hundred and sixty-two hospitalized IBS patients were treated with either a placebo or *B. infantis* at a dose of 1 × 10^6^, 1× 10^8^, or 1× 10^10^ cfu/mL for four weeks. IBS symptoms including abdominal pain or other discomfort were monitored and recorded daily. Results showed that *B. infantis* 35624 at a dose of 1 × 10^8^ cfu can effectively relieve many of the symptoms of IBS [55]. Seventy-seven IBS patients were randomized to receive either *L. salivarius* UCC4331 or *B. infantis* 35624 at the dose of 1 × 10^10^ live bacterial cells daily. They demonstrated that *B. infantis* 35624 significantly reduced symptom scores with less abdominal pain or discomfort, less bloating or distention, and better bowel movement than the placebo. In addition, the abnormal IL-10/IL-12 ratio, which is related with IBS, was also normalized by *B. infantis* 35624 supplementation [56]. Forty-four patients with Rome II positive IBS were enrolled in another 12-week clinical trial, where they were randomized to receive either 3.5 g⁄day prebiotic, 7 g⁄day prebiotic, or 7 g⁄day placebos. Results showed that galactooligosaccharide acted as a prebiotic via stimulating gut bifidobacteria in IBS patients, and effectively alleviated IBS symptoms [57]. Because several important symptoms including pain and diarrhea overlap in IBD and IBS, hopefully these interventions will provide references to use prebiotics for IBD treatment or adjuvant treatment. 

### 3.9. Gums

Partially hydrolyzed guar gum (PHGG), a water-soluble NSP produced from guar gum beans through enzymatic hydrolysis, ameliorated colonic damage and decreased MPO activity, TNF-α protein, and mRNA expression in the colonic mucosa in a TNBS-induced colitis model. The intestinal microbiota analysis found that the quantities of *Clostridium coccoides*, *Clostridium leptum*, and *Bacteroides fragilis* increased markedly in the PHGG-fed mice. Moreover, the caecal content of several SCFAs increased significantly in the PHGG-fed mice [58]. Similarly, another study proved that the supplementation of a gum mixture (guar gum and PHGG) significantly reduced the clinical score of mice with dextran sulfate (DSS)-induced colitis compared to the DSS group (*p* < 0.01) [59]. As for humans, 116 children were included in a clinical trial to study the effect of a special diet composed of comminuted chicken and PHGG, in which 57 received the special diet and 59 received the control diet. In the study group, the diarrhea of 84% was resolved compared to only 62% of the control group. In addition, the duration of diarrhea also reduced in children receiving the study diet [60]. The biscuits containing PHGG and fructo-oligosaccharides (FOS) at a dose of 6.6 g FOS and 3.4 g PHGG daily also showed prebiotic effect in a human volunteer study [61]. The stool consistency and abdominal pain along with irritable bowel syndrome were also alleviated by PHGG [62]. These studies imply that the ingestion of PHGG may be promising in the development of therapeutics for IBD.

### 3.10. Pectins

A study investigated the protective effects of wild jujube (*Zizyphus spinosus Hu*) pulp polysaccharides (WJPs) against an experimental IBD model that was induced by intrarectal administration of TNBS. WJPs are acidic heteropolysaccharides, which consist of about 40% arabinose glucose, 20% arabinose, 20% galacturonic acid, and remainder of galactose. The results showed that WJPs could ameliorate the colitis markedly, which was proved by reduced weight loss, disease activity index scores, and mucosal damage in colitis rats. Besides, the inflammatory response was partially inhibited by WJPs through the downregulation of TNF-α, IL-1β, and IL-6 expressions, and MPO activity. The effect exerted by WJPs on transepithelial electrical resistance (TER) and FITC-conjugated dextran permeability in Caco-2 cells stimulated with TNF-α further demonstrated that its protective properties on the colon in colitis was associated with barrier function by the upregulation of AMP-activated kinase (AMPK) activity [63]. Calcium pectate (CP), which is composed of mixture of 67.3% anhydrogalacturonic acid and 38 mg/g calcium, was used to study the gastroprotective effect. Results suggested that the administration of CP (39.3 kDa) prepared from citrus at doses of 25 and 50 mg/kg significantly decreased the mean area of lesions and ulcers in mice, and this protective effect was even better than traditional anti-ulcer drugs, such as fomotidine [64].

### 3.11. Modification of NSPs

NSP modification can affect the physical and chemical properties. There is growing interest in the study of the modification of different polysaccharides due to their beneficial effects. Jiang et al. [65] reported that carboxymethylation modification of *Plantago asiatica* L*.* seeds’ polysaccharides could enhance their immune activity and dendritic cell (DC) maturation, indicating that the carboxymethylated polysaccharide could be potentially applied as an immuno-enhancement therapeutic agent. According to Pomin’s suggestion, sulfated glycans, sulfated fucans (SFs), and sulfated galactans (SGs) from marine plants may be used in IBD therapies because of their highly efficient anti-inflammation and antimicrobial activities in infections [66].

Different NSPs from varied sources that have been used in IBD treatments are summarized in Table 1. It is suggested that NSPs have protective properties on intestinal diseases, especially IBD, in spite of a few reports that NSPs showed no beneficial or even a pejorative effect on IBD. However, a simple conclusion that NSP supplementation has protective or adverse effects on IBD cannot be drawn from these results, given that an NSP is a heterogeneous substance composed of group of chemicals with various properties. There are several other factors affecting the results of NSP intervention studies. The type and source of NSPs, different composition and molecular weight, the dosage, and the duration of the intervention are recognized to be important factors. Furthermore, whether the patients have UC or CD, whether the disease is active or in remission, and whether they had a damaged or intact colon may also have great influence on the results. In addition, even using the same type and dosage of NSP to treat patients of the same stage, the different interventions may still exhibit different results because the patients may have different genetic susceptibilities. It is reported that approximately 25% patients with CD have been shown to have an increased genetic risk for CD [67]. It is thus reasonable that NSPs have failed to generate consistent disease alleviation in IBD patients. Certain types of NSPs in certain patients, at certain times may even exacerbate IBD, such as when increased fermented gas further accelerates the perforation of severe UC cases. This may be an adverse effect of many easily fermentable NSPs, especially fructans. However, these side effects cannot deny the beneficial effect of NSP supplementation for IBD patients. On the contrary, it suggests that we should pay more attention to the individual differences, stages of disease, and the compositions of NSPs in the intervention trials; in particular, investigating the cause of contrary results will promote us to understand the precise mechanism of supplementing NSPs in IBD treatment. 

## 4. Mechanisms of NSPs in the Interventions of IBD

**Figure 2:**
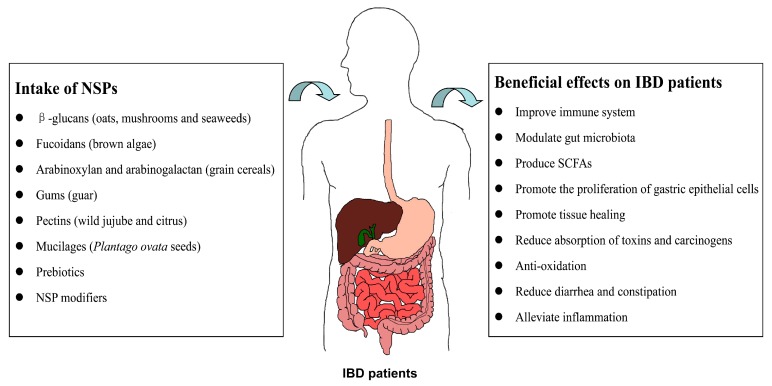
The possible positive effects of different NSPs on IBD patients. NSP: non-starch polysaccharide; IBD: inflammatory bowel disease; SCFAs: short chain fatty acids.

**Figure 3:**
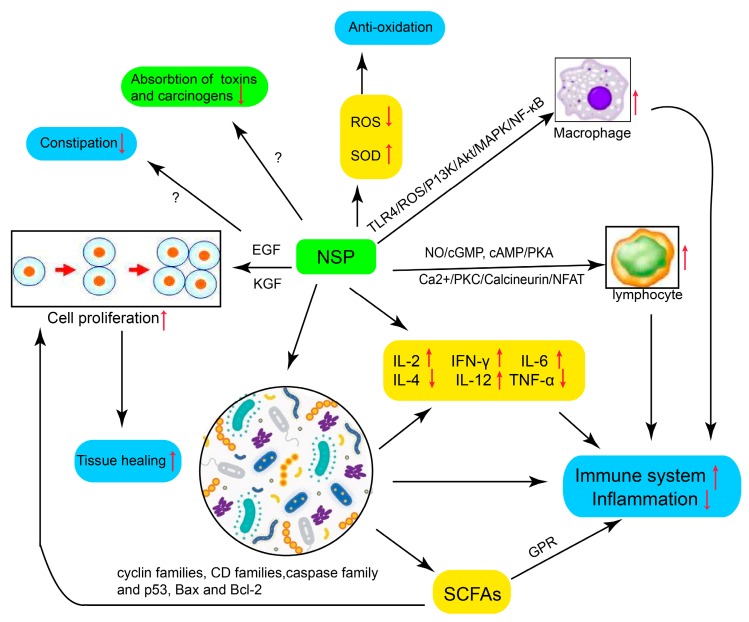
The possible molecular mechanisms of NSPs affecting IBD. NSP: non-starch polysaccharide; ROS: reactive oxygen species; SCFAs: →short chain fatty acids; SOD: superoxide dismutase; “↑”: represent “increase”; “↓”: represent “decrease”; “?”: represent “possible or unsure”.

Although great efforts have been made to study the pathogenesis of IBD, its precise etiology still remains unrevealed and is generally believed to be associated with multiple elements, including genetic background, environment, microorganism, and immunity [68]. The interaction among the disorder of the microbial constitution, the intestinal mucosal barrier, and the mucosal immune system are believed to play a vital role in the pathologic process of IBD. We summarized the possible molecular mechanisms of NSPs affecting the maintenance and recovery of IBD in the following Figure 2 and Figure 3.

### 4.1. Promote Immune System and Reduce Inflammation

Polysaccharides isolated from multiple kinds of mushrooms are proved to be immune modulators, such as *Ganoderma* and *L. edodes*. Xu et al. [69] demonstrated that glucan extracted from *L. edodes* could regulate nitric oxide (NO) production, TNF-α, and IL-6 levels in LPS-stimulated RAW 264.7 cells. Huang et al. [13] found that the immune-modulating mechanism of a polysaccharide from *Ganoderma sinense* worked mainly through multiple signal pathways: the TLR4/ROS/P13K/Akt/MAPK/NF-κB pathway in macrophages and NO/cGMP, cAMP/PKA, and Ca^2+/^PKC/Calcineurin/NFAT pathways in spleen lymphocytes. The administration of *A. sinensis* polysaccharide (AP) significantly promoted the proliferation of total spleen cells, macrophages, and T cells. The gene expression and production of IL-2 and IFN-γ were enhanced, while IL-4 was downregulated by this treatment. The differences of cytokines also led to the remarkable increase of the CD4^+^ T cell percentage in total spleen cells, plus a slight decrease of the CD8^+^ T cell proportion, which suggests that the immune-stimulating activity of AP was mainly mediated by the regulation of the expression of Th1 and Th2-related cytokines. AP also significantly increased the level of IL-12 in dendritic cells and the IFN-γ secretion of T cells in mixed lymphocyte reactions [70]. In addition, glucan extracted from mushrooms has been proved to modulate cytokine profiles and phagocyte activity, enhance protection against sepsis, infections, and inflammations, and thus exert a beneficial effect on IBD development [71,72,73,74].

The balance between T regulatory cells (Treg) and T helper cells 17 (Th-17) is broken in the DSS-induced colitis model. Oleanolic acid, which is a kind of NSP and is widely distributed in food, inhibited IL-6 and the TGFβ-induced differentiation of splenic T cells into Th17 cells. NSPs could alleviate this inflammation [75]. The oral intake of a multi-fiber mix (MF) can reduce intestinal inflammation in a DSS-stimulated colitis model through the reduction of TNF-α, IL-6 and the increase of IL-10 expression, and it also increases the number of Treg cells in the mesenteric lymph nodes. Treg percentage is correlated with the proportion of tolerogenic lamina propria-derived CD103 + RALDH + dendritic cells [76]. Dietary supplementation of chito-oligosaccharides (1% or 3%) for five months in a high-fat diet (HFD) model significantly reduced body weight, and about 25% of inflammation-related genes were modified by chito-oligosaccharides, suggesting that chito-oligosaccharide supplementation may ameliorate obesity and obesity-associated inflammation [77]. Another report showed that fucoidan induced the apoptosis of cancerous endothelial cells and downregulated the expression of the pro-inflammatory factor, indicated that it could function as an anti-inflammatory phytochemical for cancer therapy [78].

### 4.2. Modulate Gut Microbiota and Reduce Inflammation

Dysregulated gut microbiota in the gastrointestinal (GI) tract appears to be a major contributor of IBD [79]. Recent advances have implied that the genetic background may affect the composition of microorganisms harbored in the gut and exerts an impact on the individual’s susceptibility to develop IBD as well as the severity of the disease [80]. Different microorganisms colonizing in the GI tract form the human intestinal microbiome, which is involved in host digest, energy harvest, regulating immune responses, and protecting the GI tract from harmful pathogens. A healthy gut microbial environment is featured by the predominance of beneficial species, and it mainly includes *Firmicutes* and *Bacteriodes*, but also includes minor amounts of *Protobacteria* and *Actinobacteria* [81]. The intestinal disorder state is often found with excessive numbers of adverse enterobacteria and Gram-negative bacteria, and tends to cause inflammation in the GI tract. 

Some bacterial species are defined as “probiotics”, which include *Bifidobacteria*, *Lactobacillus,* and *Faecalibacterium prausnitzii*, and have immune regulatory effects. *Lactobacillus* can promote the maturation of dendritic cells, which generate interleukin (IL)-12, IL-18, and IL-23 contributing to a Th1 response, and produce IL-4 or IL-10 contributing to a Th2 response [82,83]. *Bifidobacteria* can cause an increased release of IL-10 in DC and a reduced production of IFN-γ by activated CD4^+^ T cells [84]. *Faecalibacterium prausnitzii* can increase IL-10 production and decrease serum IL-12 levels [85]. Probiotics can alleviate inflammation via reducing the expressions of inflammatory factors.

NSPs acting as prebiotics can provide a beneficial growth environment for these probiotic strains in the intestine and reduce the risk for subsequent clinical relapses of IBD [86]. Prebiotics also could inhibit potentially pathogenic bacteria, such as *Clostridium* and alleviate diarrhea, one of the important symptoms of IBD [87]. Inulin-type fructans (ITF) are naturally stored in onion, banana, chicory, and artichokes. Investigators found that ITF can promote gut health in human and animal studies [88]. In a double-blind, placebo-controlled study on obese women, ITF inhibited the growth of *Bacteroides intestinalis*, *Bacteroides vulgatus,* and *Propionibacterium,* and significantly increased the numbers of *Bifidobacterium* and *Faecalibacterium prausnitzii.* ITF can protect the intestinal barrier and remarkably decrease serum LPS levels [89]. Inulin could also inhibit in vitro intestinal colonization of *Clostridium difficile* [90], which has been proven to be a pathogen that can cause severe diarrhea, colitis, and even death [91].

The reasonable mechanisms of these beneficial effects of prebiotics include: (1) the fermentation of NSPs in the colon decreases the pH, which inhibits the growth of certain organisms such as *Bacteroides* spp. [92]; (2) some protective bacteria like *lactobacilli* and *bifidobacteria* secrete certain enzymes hydrolyzing prebiotics and lead to their own proliferation [93]; (3) some species of organisms can induce the growth of some beneficial bacteria by a cross-feeding effect. For instance, *B. longum* releases free fructose during its fermentation, which will create a beneficial environment for other organisms [94,95].

Recently, the differences of gut microbial communities contributing individual variations in cytokine responses of microbial stimulations in healthy humans were studied as part of the Human Functional Genomics Project. Stool samples and blood samples from 500 healthy individuals were taken, and six cytokine (IL-1b, TNF-α, IL-6, IL-17, IFN-γ, and IL-22) responses were measured in vivo in peripheral blood mononuclear cells. Results show that TNF-α and IFN-γ productions are strongly associated with specific microbial metabolic pathways, whereas the other four cytokines exhibit fewer, but more specific, associations with the gut microbiota [96]. Similar results were confirmed by another research team; they found that the immune responses are addressed to specific pathogens rather than specific immune pathways. There is a poor correlation between monocyte-derived and T-helper-derived cytokine responses. The group demonstrated that a strong impact of genetic heritability on cytokine production capacity after challenge with bacterial, fungal, viral, and non-microbial stimuli [97]. These findings are beyond our former knowledge and illustrate the complex relationships among gut microflora, immune response, and genetic background, which affect the pathologic process of IBD.

### 4.3. Produce SCFAs

Carbohydrates including NSPs in the colon are fermented to generate SCFAs, mainly composed of acetate, propionate, and butyrate and a number of other metabolites such as lactate, pyruvate, and ethanol, as well as the gases H_2_, CO_2_, CH_4_, and H_2_S. Butyrate enemas have showed their potential as an effective treatment for a subset of IBD patients with decreasing rectal inflammation in several studies [98,99,100]. The possible mechanisms include: (1) SCFAs are an important colonic epithelial fuel source and decrease the luminal pH, which suppresses the proliferation of pathogens and thus helps to maintain the host’s health [101]; (2) butyrate has a double effect on inflammation: Fas-mediated T cell apoptosis leads to the elimination of the inflammation source and the inhibition of IFN-γ-generated STAT1 activation, which lead to the suppression of iNOS upregulating expression in colitis [102]; (3) SCFAs can regulate gene expression through epigenetic regulation and reduces the pro-inflammatory factor expressions in human adipose tissues. SCFAs emerge as important regulators of host inflammatory responses [103,104]. SCFAs protect people from metabolic disorders via binding to G-protein-coupled receptors and altering their expressions [105,106,107]. GPR43 expresses highly in innate immune cells, especially neutrophils, and functions as an anti-inflammatory chemoattractant receptor for SCFAs. SCFAs modulate neutrophil recruitment via the dysregulated expression of G protein-coupled receptors (GPRs) during inflammatory responses [108]. A recent study found that microorganism-derived butyrate affects gut epithelial O_2_ consumption and leads to the stabilization of hypoxia-inducible factor (HIF), which is a transcription factor coordinating barrier protection [109]. Other than modulating pH, barrier function, and immune responses, SCFAs also have the abilities to regulate both cell proliferation and apoptosis through modulating proliferative genes, including cyclin families and CD families, and apoptosis genes, mainly referred to as the caspase family as well as *p53*, *bax*, and *bcl-2* [110]. Another clinical trial demonstrated that the improvement of the recovery of tissue integrity may be caused by butyrate enemas through preventing the atrophy of the colon and rectum in IBD patients [111].

### 4.4. Promote the Proliferation of Gastric Epithelial Cells and Tissue Healing

Crude polysaccharides isolated from *A. sinensis* (ASCE) were found to prevent against gastric mucosal damage in a rat model, induced by ethanol or indomethacin. In vitro studies showed that the migration of gastric epithelial cells (RGM-1) over an artificial wound was significantly promoted by ASCE. And this extract also promoted the incorporation of ^3^H-thymidine in RGM-1 cells in correlation with the dose, and increased the mRNA level of epidermal growth factor (EGF). These results strongly implied that ASCE exerts a direct healing effect on gastric mucosa wounds, partially through EGF modulation [112]. They found that ASCE could promote ulcer healing and inhibit angiogenesis, along with a significant enhancement of mucus synthesis [113]. Zhao et al. [114] found that the proliferation of intestinal intraepithelial lymphocytes and Payer’s patch cells were also increase by *Ganoderma* polysaccharide. Deters et al. [115] proved that polysaccharides isolated from *Plantago ovata* seed husks promote the proliferation of human epithelial cells via enhanced keratinocyte growth factor (KGF) using HaCaT cells.

### 4.5. Reduce Absorption of Toxins and Carcinogens

It is well known that dietary fibers can effectively reduce the absorption of carcinogens. Dietary fibers not only enhance laxation and fecal bulking, and also bind the toxins and carcinogens in the intestine [116]. Theoretically, such properties might due to a more rapid movement through the colon and thus dilute the concentration of carcinogens in the intestine. However, experiments have shown that only the NSP could reduce carcinogen bioavailability, while both NSPs and resistant starch can promote laxation and fecal bulking. On the contrary, other investigators showed that resistant starch can significantly enhance the carcinogen bioavailability [117]. The specific working mechanism needs further investigation. One speculated explanation hypothesizes some special spatial structure of NSP that allows it to wrap around the carcinogens. One study even found that NSP might enhance the apoptotic deletion of intestine cells suffering from carcinogens, thus promoting the health of the intestine [118].

### 4.6. Anti-Oxidation in the Process of Inflammation

Like other inflammatory disorders, reactive oxygen species play an important role in the progression of IBD. Polymorphonuclear cells and macrophages produce more ROS, such as superoxide and hypochlorite during the pathologic process of IBD. ROS is involved in antimicrobial activity in the lesion, but it can also cause the injury of epithelial cells in the intestine [119]. Based on this mechanism, there have been some trials on IBD therapy with dietary antioxidants [120]. In an LPS-stimulated inflammation model, rats were fed with a control diet or a diet supplemented with low and high molecular weights oat β-glucan. The results showed that high molecular weight β-glucan supplementation inhibited lipid oxidation and inflammation [121]. Wilczka et al. [122] also reported similar results; they showed that rats fed with β-glucan inhibited oxidation and inflammation in an LPS-induced enteritis model, and the oral administration of β-glucan ameliorated antioxidative potential markers like superoxide dismutase (SOD), etc.

### 4.7. Reduce Constipation

Dietary fiber has been well recognized for its bulking effect and acceleration of gut transit. *Plantago ovata* polysaccharides can absorb water, leading to augmented bulk and moisture in the stool and thereby it can function as a laxative. The increased bulk promotes normal peristalsis and bowel motility, which has clinical benefits to constipation and IBD patients [123]. *D. candidum*, a mainly water-soluble polysaccharide, was used to study the anti-constipation effect. The results showed that serum levels of motilin, gastrin, endothelin, acetylcholinesterase, substance P, and vasoactive intestinal peptide were significantly increased and the serum level of somatostatin was reduced [124]. 

## 5. Future Perspectives

Other than direct effect of NSPs on IBD, as well as other intestinal diseases, polysaccharides such as pectin, dextran, gum, alginate, inulin, and konjac glucomannan are used as the packages for oral colon-targeted drug delivery, which has attracted considerable attention because this method could increase the bioavailability of the drug at the target site and meanwhile reduce the side effects. 5-ASA, an effective anti-inflammatory drug for IBD, can be absorbed rapidly in the small intestine and its curative effect will be intensified by the polysaccharide-packaged delivery. Microspheres of chitosan with medium molecular weight (Mw) and 1:1 core/coat has been developed to achieve this goal, which has showed near 80% release of drug in the colon [125]. Chang et al. [126] developed a kind of pH-sensitive nanosphere for drug delivery to inflammatory tissues of the colon with modified sodium alginate, and the drug released in pH 6.0 buffer from drug-loaded nanospheres exhibited an obvious increase, indicating that the nanospheres may be used for colon-specific drug delivery. 

As for the direct effect of NSPs on IBD, accumulating evidences suggest that a diet lacking sufficient amounts of fiber can result in an altered gut microbiota composition and a distorted state of intestinal wellbeing, contributing to the susceptibility to local and systemic inflammatory diseases. A recent study revealed the mechanism of dietary fiber (DF) protecting the intestinal mucus barrier. During chronic or intermittent dietary fiber deficiency, the gut microbiota resorts to host-secreted mucus glycoproteins as a nutrient source, leading to the erosion of the colonic mucus barrier, which means gut mucus will get thinner and more susceptible to pathogens [127]. Taking into account the anti-inflammatory, immune stimulating, prebiotical, and healing properties of several of NSPs, we think it is necessary to add certain amount of NSPs to the diet in the early stage of IBD and post-hospital IBD patients. For patients with active forms of the diseases, especially when ulcers occur, diets with low, easily fermentable NSPs are recommended, which is referred to as a low FODMAP diet [128]. 

Overall, most studies showed that dietary NSPs were associated with beneficial effects of IBD patients, and the association were not entirely consistent. The mixed data may be related to different source of NSPs, different molecular weight of NSPs, different stages of IBD, different dosages, different genetic backgrounds, etc. Further investigation of the crosstalk between the immune cells, epithelial cells, gut microorganisms, and different kinds and molecular weight of NSPs will promote a deeper understanding of their mechanisms and will aid in the development of suitable intervention methods. We believe NSPs will be used as adjuvant therapy of IBD or should be added to the diet of IBD patients in the near future. They also can be an agent added into the diet to help people prevent the attack of IBD.

## Figures and Tables

**Table 1:** Different NSPs from various sources related with IBD.

Types of NSPs	Sources	Structure	Ref.	Experimental Method	Main Results
glucan	oat	β-1,3-and 1,4-glucan	[21]	25 g/day bran, 39 ulcerative colitis (UC) patients	high bran intake is of less value in maintaining clinical remission
			[22]	60 g/day, quiescent UC, 3 months	no patient showed signs of colitis relapse
			[23]	1% of G1 or G2 β-glucan (βG), chronic (lipopolysacchride) LPS -induced enteritis rats	different type blood leucocyte ↓
			[24]	500/1000 mg per kg β-glucan (βG), dextran sulfate (DSS)-induced colitis rats	clinical symptoms ↓; pro-inflammatory factor ↓
	mushroom	α- and β-d-glucan	[26]	60 mL/day of AndoSan, 11 Crohn’s disease (CD) and 10 UC, 12 days	inflammatory cytokine ↓; calprotectin of UC ↓
			[27]	DNA damage of lymphocytes from inflammatory bowel disease (IBD) patients under H_2_O_2_ in vitro were measured	oxidative stress in lymphocytes ↓
			[28]	oral daily intake of AndoSan, 50 patients with symptomatic UC and Crohn’s disease (CD)	marginally anti-inflammatory effects
			[29]	500/1000 mg per kg βG, DSS-induced colitis rats, LPS-stimulated RAW264.7 cell	inflammatory cytokine ↓; phosphorylation of Elk-1 at Ser84, phosphorylation of PPARγ at Ser112 ↓
			[30]	2 or 20 mg per mouse daily, DSS-induced colitis mouse	anti-inflammatory cytokines ↓; clinical symptoms ↓
			[31]	-	hepatic CYP1As expression ↓; NF-κB ↑
			[32]	SW 480 human colorectal cancer cell	proliferation ↓; diphenyl-picryl hydrazyl (DPPH ) radicals ↓
			[33]	HT-29 colon cancer cells	proliferation ↓; pro-apoptotic molecules Bax and cytosolic cytochrome-c ↑
			[35]	human colon cancer DLD-1 cells	proliferation↓, DPPH radicals ↓
	seaweed	β-1,3/1,6-glucan	[36]	Th17-major contributor to pathology of IBD measured in pig colon	expression of Th17-related cytokines (IL-17a, IL-17F, and IL-22), receptor IL23R, and IL-6 ↓
			[37]	laminarin and fucoidan, DSS-challenged pigs	body weight ↑; IL-6 ↓; *Enterobacteriaceae* ↓
	bacterial glucan	β-(1,3)-glucan	[38]	2.5 or 5 mg/kg for 2 weeks, DSS-induced IBD in mice	recruitment of macrophages ↓; expression of pro-inflammatory cytokines↓; recovery of Tregs ↑
	root of *Angelica sinensis*	α-1,3-glucan, α-1,6-glucan and other linear α-glucan	[39]	400 and 800 mg/kg, 2,4,6-trinitrobenzenesulfonic acid (TNBS) and ethanol induced colitis rat	migration of gastric epithelial cells ↑; epidermal growth factor (EGF) ↑; ulcer healing ↑; myeloperoxidase (MPO), malondialdehyde (MDA) and nitric oxide (NO) ↓; TNF-α, IL-10 & IL-2 ↓; TGF ↓; superoxide dismutase (SOD) activity ↓
fucoidan	brown algae	acidic and sulfated macromolecules (l-fucose mannose, galactose and xylose)	[42]	DSS-induced colitis mice	symptoms of colitis ↓; colon and spleen weight ↓; pathology in colon ↓; 15 pro-inflammatory cytokines ↓
mucilage	*Plantago ovata* seeds	β-l,4-and β-l,3-1inked d-xylose as backbone and arabinose, rhamnose and galacturonic acid as side chains	[45]	105 patients with UC in remission were randomized into groups to receive *Plantago ovata* seeds (10 g, twice injections per day), mesalamine (500 mg, thrice injections per day). and both at the same doses	*Plantago ovata* seeds might be as effective as mesalamine to maintain remission in UC
			[46]	5% *Plantago ovata* seeds, 13 weeks, HLA-B27 transgenic rats	colonic inflammation ↓; pro-inflammatory mediators ↓; short chain fatty acids (SCFAs) ↑
			[47]	5% *Plantago ovata* seeds, TNBS model of rat colitis	intestinal cytoarchitecture ↑; TNF-α ↓; NO synthase activity ↓
arabinoxylan arabinogalactan	grain cereal	xylans or galactans as backbone and arabinose or pentosans as side chains	[48]	21 patients with mildly to moderately active UC, 20–30 g, 24 weeks plus regular drug	clinical activity index ↓; no side effects related to (germinated barley foodstuff) GBF were observed
			[48]	high molecular weight arabinoxylans, high-fat diet mice, 4 weeks.	caecal bifidobacterial ↑; prebiotic properties
			[50]	wheat bran and resistant starch, 8 weeks, UC in remission	better gut transit, *Akkermansia muciniphila* ↓; diversity within *Clostridium cluster XIVa* ↑
prebiotics	—	oligosaccharides	[52]	xylooligosaccharides/pectin, in vitro fermentation, human fecal microbiota	SCFAs ↑; pH ↓; health-promoting bacteria ↑
			[53]	VSL#3, active UC	TLR-2 ↓; IL-10 ↑; IL-12 ↓; 10/14 patients showed a clinical response, similar to corticosteroids treatment
			[54]	chondroitin sulfate and prebiotics (resistant starch, β-glucan and mannaoligosaccharides), canine IBD, 180 days	IBD activity index ↓; histologic score ↓; serum cholesterol and paraoxonase-1 ↑
			[55]	*B. infantis* 35624, dose of 1 × 10^6^, 1 × 10^8^, 1 × 10^10^ cfu/mL, 4 weeks, women with irritable bowel syndrome (IBS)	dose of 1 × 10^8^ cfu was best to alleviate symptoms, no significant adverse events
			[56]	77 IBS patients, *Lactobacillus salivarius* UCC4331 or *B. infantis* 35624, dose of 1 × 10^10^, 8 weeks.	*B. infantis* 35624 showed a greater reduction in symptom scores and improved IL-10/IL-12 ratio
			[57]	44 Rome II positive IBS, 12 weeks, 3.5 g/day or 7 g/day prebiotic	fecal bifidobacterial ↑; 3.5 g⁄days significantly changed stool consistency and bloating, etc.
gum	guar (partially hydrolyzed)	mannose as backbone with random substitutions of galactose in a ratio of 1.6:1	[58]	partially hydrolyzed guar gum (PHGG), murine TNBS-induced colitis model	colonic damage ↓; MPO and TNF-alpha protein↓; *Clostridium cluster XIVa* and *IV* and *Bacteroides fragilis* ↑
			[59]	guar gum and PHGG, murine model of DSS-induced colitis	clinical score ↓; occludin and claudin 3, 4, and 7↑; fecal SCFAs ↑
			[60]	PHGG+ comminuted chicken diet, persistent diarrhea in 116 children, 7 day	Diarrhea ↓
			[61]	PHGG and fructo-oligosaccharides (FOS), 21 days, human	Bifidobacteria ↑
			[62]	IBS	stool consistency ↑; abdominal pain ↓
pectin	wild jujube	acidic heteropolysaccharides, about 40% of arabinose glucose, 20% arabinose,20% galacturonic acid and the rest galactose	[63]	wild jujube polysaccharides, TNBS-induced colitis rats	colitis severity ↓; mucosal damage ↓; inflammatory response ↓; AMP-activated protein kinase (AMPK) activity ↑
	citrus	calcium pectate: 67.3% anhydrogalacturonic acid and 38 mg/g calcium	[64]	25 and 50 mg/kg calcium pectate, ethanol-induced ulcers, prednisolone induced injury and H. Shay ulceration in 114 mice, 7 days	mucosa damage ↓
NSP modifiers	*Plantago asiatica*	carboxymethylation of the seeds polysaccharides	[65]	dendritic cells in vitro	MHCII ↑; IL-12, CCR7 and CXCR4 ↑; endocytosis activities ↓; mixed lymphocyte reactions ↑
	marine plants	sulfated fucans and sulfated galactans	[66]	—	anti-inflammatory activity ↑; antimicrobial activity ↑

“↑”: represent “increase”; “↓”: represent “decrease”.

## Abbreviations

IBD	inflammatory bowel disease
UC	ulcerative colitis
CD	Crohn’s disease
NSP	non-starch polysaccharide
DF	dietary fiber
SCFAs	short chain fatty acids
CRC	colorectal cancer
MDA	malondialdehyde
MPO	myeloperoxidase
PCNA	proliferating cell nuclear antigen
DSS	dextran sulfate
Treg	regulatory T cells
NK cell	natural killer cell
HLA-B27	human leucocyte antigen-B27
TNBS	trinitrobenzenesulfonic acid
GB	germinated barley
DAI	disease activity index
WB	wheat bran
RS	resistant starch
ISAPP	International Scientific Association for Probiotics and Prebiotics
TLR	toll-like receptor
IBS	irritable bowel syndrome
PHGG	partially hydrolyzed guar gum
WJPs	wild jujube polysaccharides
TER	transepithelial electrical resistance
AMPK	adenosine 5′-monophosphate activated protein kinase
FODMAPs	fermentable, oligo-, di-, mono-saccharides and polyols
TNF-α	tumor necrosis factor α
IL-1	interleukin-1
AP	*A. sinensis* polysaccharide
IFN-γ	interferon-γ
Th-	T helper cell
HFD	high-fat diet
GI	gastrointestinal
DC	dendritic cell
ITF	inulin-type fructans
GPRs	G-protein-coupled receptors
Fas	APO-1/CD95
EGF	epidermal growth factor
KGF	keratinocyte growth factor
